# Blood-based circulating biomarkers for prediction of immune-checkpoint inhibitors efficacy in renal cell carcinoma

**DOI:** 10.37349/etat.2024.00271

**Published:** 2024-09-20

**Authors:** Loubna Omri, Marie Naigeon, Ronan Flippot, Javier Gavira-Díaz, Jesus Poveda-Ferriols, Dan Nguyen, Chaimae Abdi, Alvaro Arroyo-Salgado, Nathalie Chaput, Guillermo de Velasco, Laurence Albigès, Lucía Carril-Ajuria

**Affiliations:** University of Salford, UK; ^1^Department of Medical Oncology, National Institute of Oncology, Rabat X4FH+66, Morocco; ^2^Medical Oncology Department, Centre Hospitalier Universitaire Brugmann, 1020 Brussels, Belgium; ^3^Laboratory of Immunomonitoring in Oncology, Gustave Roussy, 94805 Villejuif, France; ^4^Paris-Saclay University, School of Pharmacy, 91190 Orsay, France; ^5^Medical Oncology Department, Institut Gustave Roussy, 94805 Villejuif, France; ^6^Medical Oncology Department, Centre Hospitalier Universitaire Saint-Pierre, 1000 Brussels, Belgium; ^7^Medical Oncology Department, University Hospital 12 de Octubre, 28041 Madrid, Spain

**Keywords:** Renal cell carcinoma, circulating biomarkers, immune-checkpoint inhibitors, liquid biopsy

## Abstract

Immune checkpoint inhibitors (ICI)-based combinations have become the standard first-line treatment for advanced clear cell renal cell carcinoma (ccRCC). Despite significant improvements in survival and the achievement of sustained long-term responses, a subset of patients remains refractory to ICI, and most will eventually develop resistance. Thus, identifying predictive biomarkers for ICI efficacy and resistance is essential for optimizing therapeutic strategies. Up to now, tissue-based biomarkers have not been successful as predictive biomarkers in RCC. Circulating blood-based biomarkers offer a promising alternative. These biomarkers, including circulating immune cells, soluble factors, tumor-derived markers, and those based on metabolomics, are less invasive, offer reproducibility over time, and provide a comprehensive assessment of tumor biology and patient immune status, as well as allow dynamic monitoring during treatment. This review aims to evaluate the current evidence on the different candidate circulating biomarkers being investigated for their potential to predict ICI efficacy in RCC patients.

## Introduction

Renal cell carcinoma (RCC) accounts for around 2% of all adult malignancies [1]. It comprises different histological subtypes with different molecular characteristics, biological behavior, and response to systemic therapies [2]. Clear cell RCC (ccRCC) is the most frequent histologic subtype representing around 70–80% of all RCC [3]. At diagnosis almost 30% of cases are metastatic and around 30% will recur after surgery, and will probably require systemic therapy [4, 5].

The treatment landscape of advanced ccRCC has significantly changed during the last decade with the incorporation of immune checkpoint inhibitors (ICI) to the treatment armamentarium of advanced ccRCC. Combinations of ICI with either another ICI or an anti-angiogenic (AA), have significantly improved survival outcomes, becoming the new standard of care in first-line setting [6–14].

Despite this, there is still a notable proportion of patients that will be refractory to ICI while another subset will eventually develop resistance to ICI. Additionally, ICI are associated with non-negligible toxicities. In this context, research efforts are directed towards identifying reliable biomarkers that can help determine the best treatment strategy for our patients (i.e., ICI-ICI, ICI-AA, or even ICI or AA monotherapy).

To date, tissue-based approaches have failed to identify reliable biomarkers that are predictive of response to ICI [15]. Immunohistochemical biomarkers, such as PD-L1 or tumor infiltrating cytotoxic T cells, have been extensively studied but have failed to demonstrate a predictive role in ccRCC. Gene expression signatures developed within ICI-AA combination trials have not been validated when assessed in patients treated with dual ICI in the Checkmate 214 trial [16–18].

Circulating biomarkers emerge as an attractive alternative to tissue-based biomarkers. Blood-based biomarkers allow repeated evaluations at different time points, are easily accessible and less invasive for patients, and may be able to overcome the heterogeneity associated with biopsies. In addition, they may provide more information about the host’s immune status and could be useful in determining a patient’s potential for developing effective tumor immunity.

This review provides an overview of the different blood-based candidate biomarkers currently under study for predicting response to ICI in advanced RCC (aRCC).

## Circulating immune cells

The effectiveness of ICI therapy is intricately linked to the host’s capacity to initiate an anti-tumor immune response. Consequently, the immune profile of the host theoretically impacts the efficacy of ICI. Effector immune cells, including cytotoxic T cells and natural killer (NK) cells, are essential for the effectiveness of ICIs, as they directly attack tumor cells when checkpoint pathways are inhibited. Additionally, immune cells express checkpoint molecules such as PD-1 and CTLA-4, and the levels and activation status of these molecules can impact their function and response to ICIs [19, 20]. Regulatory T cells (Tregs) can suppress immune responses and limit the effectiveness of ICIs by inhibiting effector T cells, with high levels of Tregs in tumors counteracting checkpoint blockade effects [21]. Antigen-presenting cells (APCs) like dendritic cells and macrophages are crucial for presenting antigens to T cells and initiating immune responses, thus influencing T cell activation and the overall immune response to ICIs [22, 23]. Furthermore, cytokines produced by immune cells can modulate the immune response; for instance, interferon gamma (IFN-γ) from activated T cells can enhance PD-L1 expression in tumor cells, affecting the efficacy of ICI [24]. Tumor-associated macrophages (TAMs) can exhibit pro-inflammatory (M1-like) or anti-inflammatory (M2-like) phenotypes, with the balance between these phenotypes affecting the tumor microenvironment (TME) and the response to ICIs [25]. Additionally, interactions between immune cells and endothelial or stromal cells in the TME can influence tumor vascularization and the immune milieu, further affecting ICI efficacy [26].

**Figure 1:**
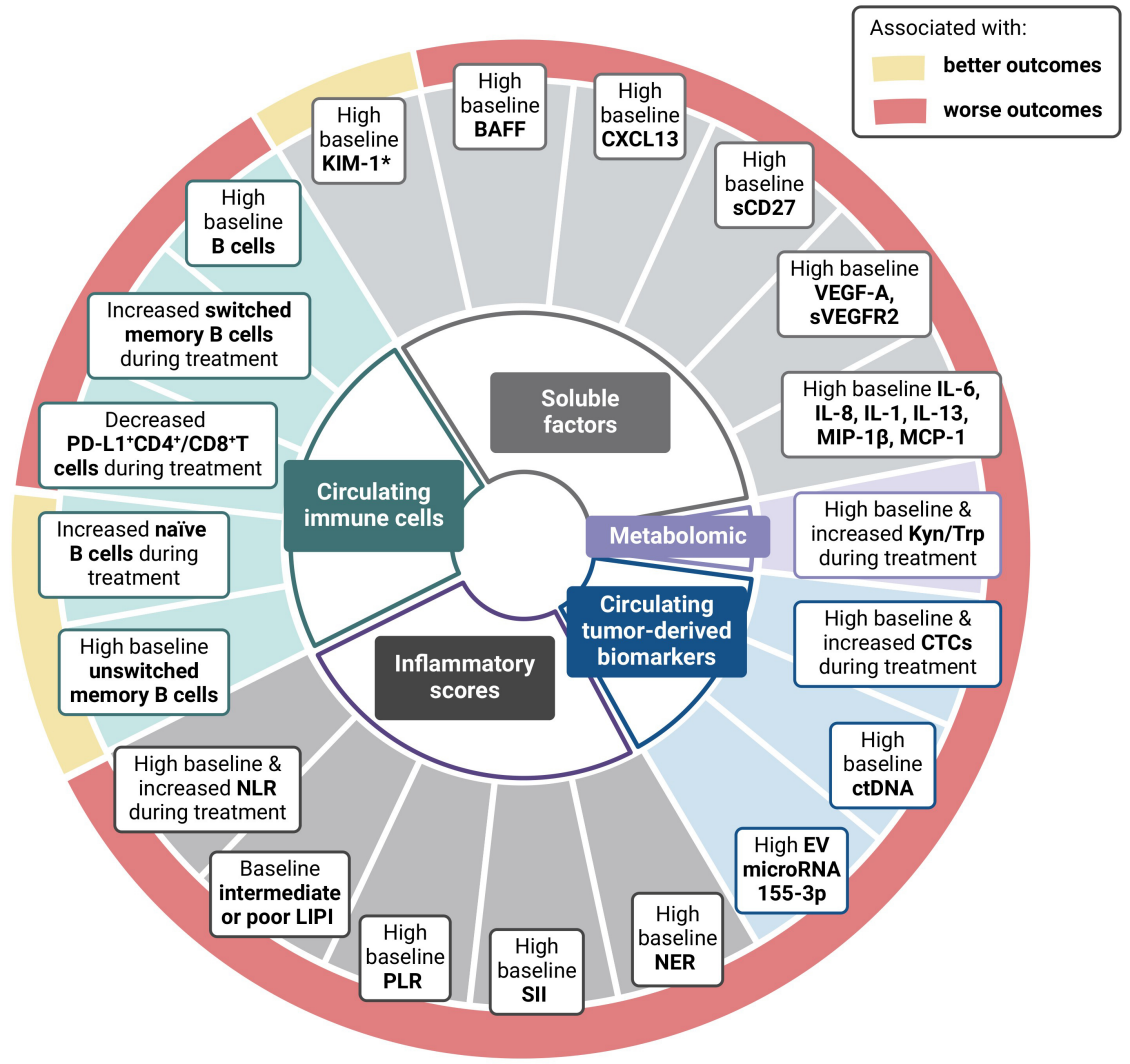
Circulating biomarkers & outcomes to immunotherapy in RCC. BAFF: B-cell activating factor; KIM-1: Kidney-Injury Molecule-1; NLR: neutrophil-to-lymphocyte ratio; LIPI: lung immune prognostic index; PLR: platelet-to-lymphocyte ratio; SII: systemic immune inflammation; NER: neutrophil-to-eosinophil ratio; EV: extracellular vesicle; ctDNA: circulating tumor deoxyribonucleic; CTCs: circulating tumor cells; Kyn: kynurenine; Trp: tryptophan; sCD27: soluble CD27

Different research groups have investigated how peripheral immune cell populations influence the response to ICI therapy across different solid tumors, including ccRCC. Infiltrating cytotoxic T cells, as primary protagonists and targets of ICI therapy, have been extensively studied; however, the results in aRCC patients remains controversial [16]. Increasing evidence highlights the role of other immune cells in the antitumor response, such as B lymphocytes [27]. Following the encouraging results of different studies supporting the role of B cells within tertiary lymphoid structures (TLS) in fostering antitumor responses across different solid tumors treated with ICI, fresh blood immune-monitoring of advanced ccRCC patients receiving nivolumab within the NIVOREN study revealed that pre-existing high levels of unswitched memory B cells (CD19^+^CD27^+^IgD^+^IgM^+^) were associated with improved clinical outcomes (*n* = 44) [overall survival (OS) HR = 0.08, *P* = 0.002 and progression-free survival (PFS) HR = 0.54, *P* = 0.048] [28] (Table 1). This B cell subset has the ability to reinitiate B cells response but also initiate a germ center reaction upon repeated antigenic stimulation [29, 30]. Interestingly, unswitched memory B cells also correlated with circulating T follicular helper (Tfh) cells, which are known to enhance B cell maturation, stimulate the expansion of T CD8^+^ lymphocytes, and with the presence of TLS. These results contrast with those reported by a pan-tumor study (*n* = 78), which included RCC patients, showing that high pretreatment levels of circulating B cells were negatively associated with response to ICI (*P* < 0.001) [31] (Table 1). The results of this study also suggest that an increased B cells frequency could identify patients at risk of progression after an initial response to ICI [31]. Finally, a small pan-tumor study, including 7 RCC tumors among the 45 different solid tumors analyzed, found that patients with increased frequency of naive B cells were more likely to benefit from ICI [disease control rate (DCR) odds ratio (OR) = 12.31, *P* = 0.039], while those with increased frequency of switched memory B cells were associated with resistance to ICI (DCR OR = 0.06, *P* = 0.025) [32] (Figure 1).

**Table 1:** Circulating immune cells and association with outcomes to immunotherapy in RCC

**Candidate biomarker**	**References**	**Year**	**Country**	***N***	**Tumor**	**Type of systemic therapy**	**Parameter level/trend indicator**	**Detection technique**	**Timepoint**	**Findings**
Unswitched memory B cells	Carril-Ajuria et al. [28]	2022	France	44	ccRCC	Nivolumab	High	Flow cytometry	Pretreatment	Improved ORR, PFS and OS
B cells	Yuan et al. [31]	2020	China	78, 12 RCC	Pan-tumor Renal carcinoma (*n* = 12, 15.19%)	ICI	High	Flow cytometry	Pretreatment	Decreased OPR Increased PD
Naive B cells	Barth et al. [32]	2022	Austria	45, 7 RCC	Pan-tumor	ICI	Increase	Flow cytometry	Pretreatment	No significant association, neither with DCR or ORR
Switched memory B cells							Increase		On-treatment changes	Improved DCR Reduced DCR
CD8, CD4^+^PD-L1^+^ T cells	Saliby et al. [33]	2023	US	60	Variant RCC	Atezolizumab plus bevacizumab	Large decrease	Flow cytometry	On-treatment changes	Worse PFS and OS

ccRCC: clear cell renal cell carcinoma; DCR: disease control rate; ICI: immune checkpoint inhibitors; ORR: objective response rate; OS: overall survival; PD: progressive disease; PFS: progression-free survival; RCC: renal cell carcinoma; SD: stable disease; US: United States

To understand the importance of circulating PD-L1^+^ T cells, a study investigated their relative changes at the third cycle of treatment (atezolizumab plus bevacizumab) compared to baseline levels prior to therapy [33] (Table 1). During treatment, all patients experienced a decrease in the percentage of CD8^+^PD-L1^+^ T cells. Patients with therapeutic resistance experienced a greater decrease in the percentage of CD8^+^PD-L1^+^ T cells compared to those who responded to therapy. This greater decrease in CD8^+^PD-L1^+^ T cells seemed to correlate with resistance. However, a similar trend was not clearly observed with the relative change in CD4^+^PD-L1^+^ T cells on-treatment. A larger decrease of PD-L1^+^ T cells on-treatment was associated with worse PFS and OS for both CD4^+^PD-L1^+^ T cells and CD8^+^PD-L1^+^ T cells.

Another small study, including 45 patients with RCC, evaluated whether the baseline diversity of the T-cell receptor β-chain (TCRB) was associated with prognosis and how the baseline and dynamic RCC tumor burdens affected the T‐cell repertoire [34]. Higher TCRB diversity was shown to be connected with an increased lymphocyte‐to‐neutrophil ratio, especially indicating elevated naive T cells. In addition, high baseline TCRB diversity in stage IV patients was associated with improved OS (HR = 0.195, *P* = 0.037).

In summary, although evidence on the value of circulating immune cell populations is limited, recent findings from small cohort studies provide promising results that warrant further investigation in larger prospective studies.

## Inflammatory routine blood markers and derived scores

Blood-based inflammatory markers could, in theory, indicate the host’s proinflammatory status and systemic immune response to cancer-related inflammation. Systemic inflammation in aRCC is associated with a poor prognosis [35, 36]. Different inflammatory routine blood parameters have been associated with worse prognosis and resistance to ICI in a variety of solid tumors, including RCC. Some of these parameters (neutrophil count, platelets, or LDH) are well-known prognostic factors in RCC and are included in established prognostic scores used in daily clinical practice, such as the IMDC (International mRCC Database Consortium) or MSKCC (Memorial Sloan Kettering Cancer Center) scores [35, 36].

The neutrophil-to-lymphocyte ratio (NLR) is one of the most studied inflammatory markers in cancer. Reflecting the balance between systemic inflammation and adaptive immune response, it has been associated with poor prognosis across different solid tumors, including RCC. This marker has been extensively studied in aRCC, with a median NLR cut-off varying between 2.5 and 5 [37, 38] (Table 2). The most robust data on the prognostic role of NLR in RCC come from a systematic review and meta-analysis by Shao et al. [38] including 6,461 RCC patients. High NLR was associated with a poor prognosis in both localized and aRCC. In addition, elevated baseline NLR in ICI-treated aRCC patients was also associated with worse OS [38]. Recent data from the JAVELIN Renal 101 trial showed a significant association between high baseline levels of NLR (cut-off: median NLR = 2.8) and inferior clinical outcomes in both the avelumab plus axitinib (low vs. high NLR OS HR, 0.51; 95% CI, 0.300–0.871) and the sunitinib arms (low vs. high NLR OS HR, 0.30; 95% CI, 0.174–0.511), continuing to support the prognostic role of NLR in patients with aRCC [39]. Moreover, Rebuzzi et al. [40, 41] developed an improved version of the IMDC score, the “MeetURO score” by including the NLR index and the presence of bone metastases, which enabled them to split RCC patients treated with ICI into five prognostic groups. Additionally, on-therapy NLR variation has also been associated with clinical outcomes to ICI. In a retrospective study including advanced non-small cell lung cancer (NSCLC) (*n* = 75) and RCC (*n* = 86) patients, any NLR increase at week 6 was associated with worse outcomes, compared to NLR decrease [42]. Similarly, Young et al. [43] found that NLR ≥ 3 after 12 weeks of ICI-based first-line therapy was associated with worse outcomes (17.5 months vs. 40.3 months, *P* < 0.001), and normalization of NLR in patients with baseline elevation was associated with superior OS (40.3 months vs. 14.7 months, *P* = 0.004) [43]. Monitoring NLR over time could help guide treatment intensification or de-escalation strategies; however, validation of these findings in prospective studies is still needed.

**Table 2:** Inflammatory routine laboratory derived scores and association with outcomes to immunotherapy in RCC

**Candidate biomarker**	**References**	**Year**	**Country**	***N***	**Tumor**	**Timepoint**	**Type of systemic therapy**	**Cut-off/Trend indicator**	**Findings**
NLR	Bilen et al. [39]	2022	US	886	RCC	Pretreatment	Avelumab plus axitinib or sunitinib	High	Worse OS and PFS
NLR	Simonaggio et al. [42]	2020	France	161	RCC and NSCLC	On-treatment changes	Nivolumab	Increase	Worse OS and PFS
NLR	Young et al. [43]	2024	UK	132	RCC	Pretreatment On-treatment changes	ICI combinations	≥ 3 at baseline	NS trend for worse OS
								≥ 3 at 12 weeks	Worse OS
								Normalization of pre-treatment elevation	Superior OS and ORR
NLR	Ishihara et al. [136]	2019	Japan	58	RCC	Pretreatment	Nivolumab	≥ 3	Worse OS after MVA, worse PFS only on UVA
NLR	Suzuki et al. [137]	2020	Japan	65	RCC	Pretreatment	Nivolumab	≥ 5	Worse OS
NLR	Shirotake et al. [138]	2019	Japan	54	RCC	Pretreatment	Nivolumab	≥ Median value (2.89)	NS
NLR	Zahoor et al. [54]	2018	US	90	RCC	Pretreatment	Nivolumab	≥ 4.2	Worse PFS
NLR	Tucker et al. [56]	2021	US	110	RCC	Pretreatment	Nivolumab plus ipilimumab	≥ 3.42	Worse OS
LIPI	Meyers et al. [47]	2019	Canada	643	NSCLC, melanoma and RCC (145, 25%)	Pretreatment	ICI	Good LIPI, 0 factor	No difference in OS, PFS or ORR between the good and intermediate LIPI groups
								Intermediate LIPI, 1 factor	
								Poor LIPI, 2 factors	Worse OS and PFS
LIPI	Carril-Ajuria et al. [48]	2024	France	1,084	ccRCC	Pretreatment	Nivolumab plus ipilimumab vs. sunitinib	Intermediate/Poor LIPI (1–2 factors) vs. good LIPI (0 factor)	Worse OS in both treatment arms
NER	Zhuang et al. [139]	2023	US	184	RCC	Pretreatment	ICI	High NER > 49.2	Worse OS No significant difference for PFS
NER	Tucker et al. [56]	2021	US	110	RCC	Pretreatment	Nivolumab plus ipilimumab	≥ Median value 26.4	Worse PFS, OS and ORR
PLR	Iinuma et al. [59]	2021	Japan	43	RCC	Pretreatment	Nivolumab plus ipilimumab	High Median 215.6	Poor PFS
SII	Iinuma et al. [59]	2021	Japan	43	RCC	Pretreatment	Nivolumab plus ipilimumab	Median SII of 730	Improved survival in the SII low

ccRCC: clear cell renal cell carcinoma; ICI: immune checkpoint inhibitors; LIPI: lung immune prognostic index; MVA: multivariate; UVA: univariate; NER: neutrophil-to-eosinophil ratio; NLR: neutrophil-to-lymphocyte ratio; NS: non-significant; NSCLC: non-small cell lung cancer; ORR: objective response rate; OS: overall survival; PFS: progression-free survival; PLR: platelet-to-lymphocyte ratio; RCC: renal cell carcinoma; SII: systemic immune inflammation; UC: urothelial carcinoma; UK: United Kingdom; US: United States. Meta-analysis not included

The lung immune prognostic index (LIPI), defined by pretreatment levels of derived NLR (dNLR) and LDH, was initially developed and assessed in lung cancer patients treated with ICI [44–46]. Subsequent studies have confirmed its association with clinical outcomes to ICI across other tumor types [44–50]. Until recently, the only evidence of the role of LIPI in aRCC relied on a multi-tumor retrospective study including NSCLC, melanoma and RCC (145, 25%) patients treated with ICI [47] (Table 2). In this study, LIPI stratification was associated with OS in aRCC patients treated with ICI (*P* < 0.005) [47]. The most robust data to date on the value of LIPI in aRCC come from a recent study evaluating its impact in three different prospective studies [NIVOREN study: nivolumab cohort; TORAVA trial: vascular-endothelial growth factor (VEGF)/VEGFR targeted therapy; and Checkmate 214 trial: nivolumab plus ipilimumab (nivo-ipi) vs. sunitinib] [48]. Initial results showed an association of LIPI stratification with worse outcomes in aRCC treated with nivolumab (LIPI-good 30.1 vs. 13.8 months in the LIPI intermediate/poor; HR, 0.47), but no associations with clinical outcomes were found in those treated with VEGF/VEGFR therapy, suggesting a potential predictive role for LIPI. However, this was not confirmed in the Checkmate 214 trial, where LIPI stratification was associated with worse survival outcomes irrespective of therapy type, whether nivolumab plus ipilimumab or sunitinib (nivo-ipi: LIPI good vs. intermediate/poor: HR, 0.55; *P* < 0.001; sunitinib: LIPI good vs. int/poor: HR, 0.38; *P* < 0.001) [51] (Figure 1). Thus, in contrast to NSCLC, LIPI appears to have more of a prognostic rather than a predictive value for response in aRCC.

Although their role in the immune antitumor response is less known, pre-clinical studies suggest tumor-associated eosinophilia may enhance antitumor response by promoting CD8^+^ T cell infiltration [52]. In the study by Simon et al. [53], higher levels of circulating eosinophils were associated with improved response to ICI, and eosinophils from ICI-treated patients were enriched for IFN-γ response signatures, which are known to be associated with benefit from ICI therapy. Based on these data, Zahoor et al. [54] conducted a retrospective study (*n* = 90) of aRCC patients treated with nivolumab. They found that patients with higher baseline eosinophil counts were associated with a lower risk of progression (HR, 0.54; *P* = 0.042). In contrast, the retrospective study (*n* = 65) conducted by Herrman et al. [55] failed to show a significant association between baseline circulating eosinophil counts and outcomes to ICI but found that patients experiencing an increase in eosinophils at six weeks of treatment were associated with an improved response to ICI. In the same line, Tucker et al. [56] showed that aRCC patients receiving nivolumab plus ipilimumab with lower baseline levels of neutrophil-to-eosinophil ratio (NER) presented improved clinical outcomes compared to those with higher NER at baseline (OS HR 0.31, *P* < 0.01) [56]. In addition, the same team evaluated NER on-treatment changes and found that patients with a decreased NER at week 6 of treatment were associated with improved clinical outcomes in double ICI-treated aRCC patients (HR: 0.67, *P*-value: 0.002) [57] (Figure 1).

Other scores, such as the platelet-to-lymphocyte ratio (PLR) and the systemic immune-inflammation (SII) index, have also been evaluated in the context of aRCC treated with ICI. High PLR has been correlated with worse outcomes to ICI across different cancer types [58]. Similarly, Iinuma et al. [59] reported an association between high PLR and worse outcomes in aRCC patients receiving nivolumab plus ipilimumab (1-year PFS, 75.5% for low PLR vs. 49.7% for high PLR; *P* = 0.034). In this study, the SII was also associated with worse PFS, which is in line with previous studies across different cancer types (*P* = 0.023) [60].

## Circulating soluble factors

Soluble factors, including cytokines and chemokines, play a critical role in anti-tumor immunity [61]. Cytokines and chemokines are released by both adaptive and innate immune cells, stromal cells, and tumor cells, and can be measured at both tissue and systemic levels. Certain soluble factors, such as IL-6, IL-8, and VEGF, are involved in carcinogenesis, myeloid inflammation, and promote immunosuppression, while others, such as the chemokine CXCL13 and the B-cell activating factor (BAFF), are involved in B cell activation, survival, and differentiation [62–64].

### a) IL-6 and IL-8

Both IL-6 and IL-8 contribute to the recruitment of myeloid-derived suppressor cells to the TME, hindering the anti-tumor activity of cytotoxic T cells, and have been associated with poor clinical outcomes across different tumor types [65–69]. Tran et al. [70] observed a negative association between baseline levels of circulating IL-8 and PFS in aRCC patients treated with AAs (*P* = 0.006).

In a large multi-tumor study conducted by Schalper and colleagues [67], higher pre-treatment levels of circulating IL-8 were associated with poor outcomes in lung cancer, melanoma, and aRCC patients receiving nivolumab, double ICI, everolimus, or docetaxel, which suggests a more prognostic than predictive role (for aRCC, nivolumab OS HR: 2.56, *P* < 0.001; everolimus OS HR: 2.40, *P* < 0.001). In a post-hoc analysis of the IMmotion150 trial, higher levels of IL-8 in plasma and peripheral blood mononuclear cells (PMBCs) were linked to a poor therapeutic response to atezolizumab and lower antigen presentation in metastatic urothelial carcinoma (UC) and metastatic RCC (mRCC) patients (plasma IL-8, HR: 2.55, *P* = 0.017), even in T cell-infiltrated tumors [67, 71] (Table 3). Recent data from the NIVOREN phase 2 study not only confirm the association between elevated baseline levels of IL-8 and poor outcomes in pretreated aRCC patients treated with nivolumab (HR = 2.57, *P* < 0.001), but also show an association between baseline levels of IL-8 and the tissue-based myeloid gene expression signature of the IMmotion150 (*P* = 0.041) [72, 73]. These results are consistent with the findings of Schalper et al. [67], indicating a positive association between circulating IL-8, tumor *CXCL8* gene expression, and tumor infiltration by neutrophils, suggesting a potential involvement of these cytokines in protumoral inflammation.

**Table 3:** Soluble factors and association with outcomes to immunotherapy in RCC

**Candidate biomarker**	**References**	**Year**	**Country**	***N***	**Tumor**	**Detection technique**	**Timepoint**	**Type of systemic therapy**	**Parameter level/trend indicator**	**Findings**
IL-8	Schalper et al. [67]	2020	US	1,344	NSCLC, melanoma and RCC	MAP immunoassay	Pretreatment	Nivolumab	High	Worse OS
IL-8	Yuen et al. [71]	2020	US	1,445	RCC and UC	Simple Plex Ella	Pretreatment	UC: chemotherapy or atezolizumab RCC : atezolizumab plus bevacizumab, atezolizumab or sunitinib	High	Worse OS in atezolizumab NS trend for worse OS in atezolizumab + bevacizumab Lower ORR but NS
IL-8	Carril-Ajuria et al. [28]	2022	France	233	RCC	MSD assay	Pretreatment	Nivolumab	High	Worse OS and PFS
IL-6	Sang et al. [75]	2022	Korea	58	RCC	Cytometric bead array assay	Pretreatment	Pembrolizumab plus axitinib	High	Worse OS and PFS
Il-6	Carril-Ajuria et al. [28]	2022	France	233	RCC	MSD assay	Pretreatment	Nivolumab	High	Worse OS and PFS
IL-1, IL-6, IL-13, MIP-1β, and MCP-1	Saliby et al. [33]	2023	US	60	Variant RCC	Luminex fluorescent bead array platform	Pretreatment	Atezolizumab plus bevacizumab	High	Worse OS and PFS
VEGF-A and sVEGFR2	Mauge et al. [140]	2021	France	200	ccRCC	NA	Pretreatment	Nivolumab	High	Worse PFS
VEGF	Carril-Ajuria et al. [28]	2022	France	233	ccRCC	MSD assay	Pretreatment	Nivolumab	High	Worse OS
VEGF-A	Saliby et al. [33]	2023	US	60	Variant RCC	Luminex fluorescent bead array platform	Pretreatment	Atezolizumab plus bevacizumab	High	Worse OS and PFS
VEGF	Choueiri et al. [78]	2021	US	886	ccRCC	NA	Pretreatment	Aveluzmab plus axitinib	High	No association
CXCL13	Carril-Ajuria et al. [28]	2022	France	44	ccRCC	MSD assay	Pretreatment	Nivolumab	High	Worse OS
BAFF	Carril-Ajuria et al. [28]	2022	France	44	ccRCC	MSD assay	Pretreatment	Nivolumab	High	Worse OS
sPD-L1	Mahoney et al. [82]	2022	US	91	ccRCC	ELISA	Pretreatment	Nivolumab	High	Worse OS
sPD-L1	Incorvaia et al. [85]	2020	Italy	56	ccRCC	ELISA	Pretreatment	Nivolumab	High	Improved PFS
sCD27	Benhamouda et al. [89]	2022	France	81	ccRCC	ELISA	Pretreatment	Nivolumab	High	Worse OS
KIM-1	Albiges et al. [91]	2024	France	778	RCC	Affinity-based proximity extension assay (PEA) High sensitivity electrochemiluminescence (ECL)	Pretreatment	Atezolizumab	High	Reduced DFS Better DFS with atezolizumab vs. placebo

ccRCC: clear cell renal cell carcinoma; DFS: disease-free survival; MAP: human multianalyte profile immunoassay platform; MSD: Meso Scale Discovery assay; NA: not available; NS: non-significant; NSCLC: non-small cell lung cancer; ORR: objective response rate; OS: overall survival; PFS: progression-free survival; RCC: renal cell carcinoma; sCD27: soluble CD27; sPD-L1: soluble PD-L1; SS: statically significant; UC: urothelial carcinoma; US: United States; KIM-1: Kidney-Injury Molecule-1; BAFF: B-cell activating factor; VEGF: vascular-endothelial growth factor

Elevated circulating IL-6 is associated with a poor prognosis in localized and aRCC, however, few studies have analyzed the association between IL-6 levels and clinical outcomes in aRCC treated with ICI [74]. A small Korean study (*n* = 58) found that in aRCC patients treated with pembrolizumab plus axitinib, those with high baseline levels of circulating IL-6 exhibited significantly inferior response rates, PFS (HR: 3.51, *P* = 0.003), and OS (HR: 7.18, *P* = 0.001) compared to those with low levels of IL-6 [75] (Table 3). Moreover, CD8^+^ T cells from patients with high baseline levels of IL-6 produced less IFN-γ and TNF-α, suggesting a less effective antitumoral immune response [75]. Carril-Ajuria and colleagues [72, 73] also observed a negative association between baseline levels of IL-6 and clinical outcomes (PFS and OS) in aRCC patients treated with nivolumab within the NIVOREN study (OS HR = 3.28, *P* < 0.001). Recently, Saliby et al. [33] characterized blood- and tissue-based biomarkers in patients with variant RCC histology or any RCC histology with sarcomatoid differentiation, and evaluated their association with the response to atezolizumab plus bevacizumab. Interestingly, baseline levels of circulating inflammatory cytokines (IL-1, IL-6, IL-13, MIP-1β, and MCP-1) correlated with one another, were enriched in poor IMDC patients, and were associated with worse PFS and OS under atezolizumab plus bevacizumab [33].

### b) VEGF

Baseline levels of soluble VEGF-A and sVEGFR2 were associated with poor survival outcomes in treatment-naive and pretreated advanced ccRCC patients receiving nivolumab in the BIONIKK and NIVOREN trials, respectively [72, 76] (Table 3). A small cohort study found no association between baseline levels of VEGF-A and sVEGFR2 and clinical outcomes in advanced ccRCC patients treated with pembrolizumab plus axitinib [77]. These findings could suggest that combining an AA with ICI might counteract the detrimental effect of high VEGF seen in ccRCC patients undergoing ICI monotherapy. However, results from different studies are conflicting, and the potential predictive role of soluble VEGF is still unclear. Thus, while Saliby et al. [33] reported a significant association between higher baseline levels of VEGF-A and worse PFS and OS in patients with advanced variant histology RCC treated with atezolizumab plus bevacizumab, this was not observed in aRCC patients receiving avelumab plus axitinib [78]. In addition, previous studies have also found a negative association between baseline levels of soluble VEGF and survival outcomes in advanced ccRCC patients treated with AAs [79, 80]. Overall, these findings would therefore suggest a more prognostic role. In the study by Saliby et al. [33], the impact of the on-treatment dynamic evolution of circulating VEGF-A on clinical outcomes was also evaluated. A higher increase in plasma VEGF-A throughout therapy, compared to baseline, was surprisingly associated with better clinical outcomes [33].

### c) B-cell related soluble factors

Following encouraging findings from different studies suggesting B cell tumor infiltration as a predictor of response to ICI across different solid tumors, including RCC, circulating B cells populations and B-cell-related soluble factors such as CXCL13 or BAFF have also been evaluated in the context of RCC and ICI. As previously mentioned in the NIVOREN study, high levels of baseline circulating unswitched memory B cells were associated with improved response, PFS, and OS in aRCC patients treated with nivolumab [28]. Interestingly, this population of B cells was negatively correlated with baseline levels of specific B-cell-related soluble factors: CXCL13 (*r* = −0.55, *P* < 0.001), a chemokine involved in the homeostatic organization of B-cell zones in secondary lymphoid tissue, and BAFF (*r* = −0.42, p=0.007), a key factor in B-cell activation [28] (Table 3). Consistently, these two soluble factors were associated with worse survival outcomes in aRCC patients treated with nivolumab (*n* = 40). These findings were validated in an independent dataset from the same study (*n* = 313; BAFF HR: 1.73, *P* = 0.002; CXCL13 HR: 1.52, *P* = 0.017) (Figure 1).

### d) Soluble PD-L1

Tumor PD-L1 is an established poor prognostic factor in aRCC; however, its role as a predictor of response to ICI is still unclear [81]. Given that PD-L1 expression can vary dynamically among both tumor and immune cells in the TME, soluble PD-L1 (sPD-L1) might offer a more accessible and representative surrogate of overall tumor

expression [75]. Building on a previous meta-analysis (*n* = 1,040) of sPD-L1 in different solid tumors, including RCC, which showed a negative association between sPD-L1 and survival outcomes, Mahoney et al. [82] analyzed serum levels of sPD-L1 in two cohorts of RCC (Checkmate 009, *n* = 91) and melanoma (Checkmate 038-Part 1, *n* = 78) patients treated with nivolumab. In the RCC cohort high baseline levels of sPD-L1 and an on-treatment increase of sPD-L1 were associated with disease progression under nivolumab (Table 3). The association of high baseline sPD-L1 with worse outcomes was confirmed in a meta-analysis (*n* = 1,076) of different solid tumor types, including RCC patients treated with ICIs [83]. It is important to note that this negative association between high baseline levels of sPD-L1 and survival has also been observed in treatment-naive advanced ccRCC patients treated with sunitinib, which supports a more prognostic role [84]. Nevertheless, the results of another small study by Incorvaia et al. [85] show an increased PFS in RCC patients treated with ICI with high baseline sPD-L1 levels compared to those with low baseline sPD-L1. Thus, the role of sPD-L1 in aRCC treated with ICI still remains controversial.

### e) Soluble CD27

CD70 is a costimulatory molecule known to stimulate CD27-expressing T cells, such as naive and central memory T cells. The interaction between CD27 and CD70 results in the release of soluble CD27 (sCD27). However, prolonged exposure to CD27-CD70 costimulatory signals can exhaust the T-cell pool and lead to depletion of naïve T cells [86–88]. Interestingly, ccRCC expresses the highest levels of CD70 among solid tumors. In a recent study by Benhamouda and colleagues [89], TME CD27^+^ T cells from ccRCC patients were associated with an apoptotic and dysfunctional signature compared to CD27^–^ T cells. In addition, intratumoral CD27-CD70 interaction correlated with sCD27. Consistently, higher baseline levels of sCD27 were associated with poor OS in ICI-treated patients (HR: 5.02, *P* = 0.004) but not in patients treated with AA therapy (*P* = 0.35), suggesting that sCD27 could serve not only as a surrogate marker of T cell dysfunction in the TME but also as a potential ICI-resistance biomarker [89] (Table 3). However, validation in larger prospective randomized clinical trials is needed before incorporation into routine clinical practice.

### f) Circulating proteins

The Kidney-Injury Molecule-1 (KIM-1), a transmembrane protein highly expressed in RCC and whose ectodomain circulates and can be detected in plasma, has also gained interest in the last years [90].

A post-hoc analysis from the ASSURE trial, which evaluated the benefit of adjuvant sunitinib or sorafenib vs. placebo in high-risk resected RCC patients, found a significant association between high levels of circulating KIM-1 post-nephrectomy and worse disease-free survival (DFS) (HR: 0.56, *P* < 0.001) and OS (HR: 0.71, *P* < 0.001) [90]. Recently, the results from an exploratory analysis of the IMmotion010 trial not only confirmed the association between high baseline levels of circulating KIM-1 and worse prognosis in high-risk resected RCC patients, but also found an association between post-nephrectomy levels of circulating KIM-1 and improved clinical outcomes with atezolizumab vs. placebo (HR: 0.72, 95% CI: 0.53–0.99) (Table 3).These results, therefore, suggest that circulating KIM-1 could be a marker of minimal residual disease (MRD) and may also behave as both a biomarker of poor prognosis and a predictive biomarker of atezolizumab efficacy in the adjuvant setting [91].

A recent cohort study including 36 ccRCC patients conducted by Lucarelli and colleagues [92] reported that tumor expressing high levels vs. those expressing low levels of the transmembrane glycoprotein mucin 1 (MUC1) were associated with an altered metabolism, higher vascularization, lower immune infiltration and higher M2-tumor associated macrophage response, and lower PD-L1 expression, suggesting these tumors would theorically derive less benefit from ICI. They also found that the soluble form of MUC1, the cancer antigen (CA) 15.3, was associated with higher nuclear grade, lymph node involvement and visceral metastases (*P* < 0.001), as well as with inferior cancer specific survival and PFS (*P* = 0.01) [92]. Although hypothesis-raising, both MUC1 and circulating CA 15.3 still need to be evaluated in the context of ICI treatment.

## Circulating tumor-derived biomarkers

The idea of measuring tumor-derived blood biomarkers is not new. For years, and in several cancer types, we have used different tumor protein markers such as CA 15.3, CA 125, prostate-specific antigen (PSA), or carcinoembryonic antigen (CEA) to detect disease recurrence, progression, and response to therapy [93]. However, these tumor markers lack specificity and are not able to predict response to specific treatment types and thus guide treatment selection. During the last decade, new tumor-derived biomarkers such as circulating tumor cells (CTCs), circulating tumor deoxyribonucleic (DNA; ctDNA) and micro-ribonucleic acids (miRNAs) have been developed. This is what we commonly refer to as liquid biopsy, and it has the potential to help us detect cancer, guide treatment selection, assess real-time tumor response to therapy, and identify resistant clones [94].

### a) Circulating tumor cells

CTCs are cancer cells that circulate in the bloodstream after being shed from primary or metastatic tumors. CTCs have been shown to be associated with prognosis across different solid tumors [95–99]. Currently, there is no standardized CTC detection method [100, 101]. Given that CTCs have been implicated in tumor metastasis and recurrence, they are difficult to detect in early-stage RCC. In a recent meta-analysis including 12 studies and 767 RCC patients, CTCs were more likely to be found in advanced than in localized disease (OR, 2.29; *P* = 0.002). Curiously, the sensitivity of CTCs in ccRCC (69%) was significantly higher than in non-ccRCC subtypes (34%) [100]. CTCs are associated with poor prognosis in both localized and aRCC [102–104]. Basso et al. [105] reported worse survival outcomes for aRCC patients receiving first-line AA therapy with ≥ 3 CTCs at baseline, although an association with response was not observed (*n* = 95). Only one study has evaluated the role of CTCs in the context of ICI. In this study, Bootsma et al. [106] profiled 457 blood samples collected longitudinally from 104 aRCC patients receiving ICI, confirming that CTC enumeration is prognostic in aRCC treated with ICI (Table 4). Moreover, an on-treatment increase in CTC levels was strongly and negatively associated with OS [106] (Figure 1). They also investigated the expression of HLA I to PD-L1 (HP ratio) by CTCs. Interestingly, the HP ratio decreased over time in patients treated with ICI, raising the hypothesis that tumor cells with high HLA I and low PD-L1 would be more likely to be cleared by ICI. Additionally, if a patient’s HP ratio does not drop during ICI therapy, it could suggest a poor response [106].

**Table 4:** Circulating tumor-derived biomarkers and association with outcomes to immunotherapy in RCC

**Candidate biomarker**	**References**	**Year**	**Country**	***N***	**Tumor**	**Timepoint**	**Type of systemic therapy**	**Parameter level/trend indicator**	**Detection technique**	**Findings**
Circulating tumor cells	Bootsma et al. [106]	2022	US	104	RCC	Pre-treatment	ICI	High	Nikon Ti-E microscope with automated XYZ stage	Worse OS
						On-treatment		Increase		
Circulating tumor DNA	Maia et al. [119]	2017	Brazil	34	RCC	Pretreatment	Different types of systemic therapy, including ICI		College of American Pathology-accredited comprehensive plasma assay	No significant associations
Circulating tumor DNA	Chehrazi-Raffle et al. [120]	2023	US	12	RCC	Pretreatment	ICI	Median VAFs	TARDIS	Distinguished those achieving PR (0.181%) from those with CR (0.007%)
EV microRNA-155-3p	Soleimani et al. [126]	2024	Canada	40	RCC	Pretreatment	-Ipilimumab plus nivolumab -Pembrolizumab plus axitinib -Avelumab plus axitinib		exoRNeasy serum/plasma midi kit Applied Biosystems TaqMan assays TaqMan microRNA RT kit + TaqMan miRNA assay	Lower levels in responders

CR: complete response; ICI: immune checkpoint inhibitors; OS: overall survival; PR: partial response; RCC: renal cell carcinoma; US: United States; EV: extracellular vesicle; miRNAs: micro-ribonucleic acids

### b) Tumor nucleic acids

Extracellular tumor DNA, also known as cell-free DNA (cfDNA), and ctDNA, which represents the portion of mutated cfDNA derived from cancer cells, can also be detected in plasma samples [107]. Various studies have demonstrated the diagnostic utility of cfDNA. For instance, Feng et al. [108] observed significant differences in cfDNA levels between RCC patients and healthy controls, noting correlations with tumor stage, grade, and metastatic burden. Additionally, cfDNA methylation has also shown promising results [109–111]. Conversely, the detection of ctDNA in renal cancer is lower than in other cancers, which limits its use as a diagnostic tool [112, 113]. However, results of a recent Korean study (*n* = 48) suggest that ctDNA could predict pT3a upstaging in cT1a ccRCC tumors [114]. Moreover, results from a recent study assessing MRD in the adjuvant setting with 61 RCC patients suggest ctDNA, although not ready for primetime, could have the potential to guide adjuvant treatment [115]. In this study, ctDNA negative patients in the non-adjuvant cohort had a negative predictive value of 92%. Additionally, several studies have identified cfDNA levels as prognostic markers in localized RCC [110, 116]. Yamamoto and colleagues [117] confirmed the prognostic role of cfDNA levels in both localized and mRCC patients. Regarding the role of cfDNA as a predictor of response to systemic therapy, data are still scarce with only a few small cohort studies. Feng et al. [108] observed that cfDNA decreased in RCC patients responding to sorafenib, while cfDNA levels increased in non-responders. In the study by Yamamoto et al. [117], persistence of detectable DNA was associated with an inferior response to AAs. Similarly, cfDNA levels at baseline were associated with a higher response and improved PFS/OS in RCC patients receiving either ICI or TKI [118]. Conversely, no significant associations between ctDNA levels and OS were observed in RCC patients treated with different types of systemic therapy [119]. Recently, Chehrazi-Raffle et al. [120] tested a novel ultrasensitive DNA assay (TARDIS, targeted digital sequencing) in 12 RCC patients undergoing ICI-based therapy (either nivolumab or nivolumab plus ipilimumab) with promising results (Table 4). TARDIS was able to distinguish those achieving partial response (PR) from those achieving complete response (CR), and to prospectively identify patients with subsequent progression [120]. Finally, several studies have demonstrated a low gene alteration (GA) concordance between ctDNA and tumor tissue sequencing, which raises concerns about its potential use to guide treatment selection [118].

miRNAs are small non-coding RNAs that play a key role in regulating gene expression [121]. Although several studies have investigated the role of miRNAs in RCC detection, to date, there is only one study, with reported results evaluating the role of miRNAs as predictors of response to ICI in aRCC [122–126]. In this study, Soleimani et al. [126] investigated the presence of immune-specific extracellular vesicle (EV) miRNAs in the plasma of aRCC patients before ICI initiation. miRNA-155-3p was significantly lower in responders compared to non-responders (Table 4; Figure 1). These results suggest that miRNA-155-3p could be a predictor of response to ICI in RCC, and are consistent with those reported in melanoma patients treated with ICI [126–128]. Interestingly, in another study constructing a four-miRNA model for RCC screening, miRNA-155-5p was able to distinguish between RCC patients and normal controls, while also displaying a significant association with prognosis [129]. Although these data warrant further validation, they are also supported by a biological rationale. miRNA-155-3p is the result of the *MIRHG155* gene, also known as the “B-cell Integration Cluster (*BIC*) gene” or “Master regulator of inflammation”, due to its role in modulating the inflammatory response and its critical implication in the diversification of the antibody repertoire [130]. This is consistent with the emerging evidence supporting the role of B cells in the antitumor immune response.

Overall, liquid biopsy is a non-invasive and repeatable tool that allows to monitor the dynamic evaluation of tumors. Although detection rates are low, recent studies have shown promising results suggesting further investigation of liquid biopsy components such as cfDNA, ctDNA, and EV miRNAs. These studies could potentially help us guide treatment selection and decisions regarding treatment de-escalation or intensification in aRCC.

## Metabolomics

The tryptophan-kynurenine-aryl hydrocarbon receptor (Trp-Kyn-AhR) pathway contributes to immunosuppression in T cell inflammed tumors [131]. Kyn results from Trp catabolism by indoleamine 2,3-dioxygenase (IDO) or trytpophan 2,3-dioxygenase (TDO) [132]. Trp degradation and depletion contribute to tumor evasion, while increasing Kyn metabolites which contribute to immunosuppression and cancer progression [132]. Despite promising results in previous preclinical studies showing that suppression of this pathway could enhance ICI efficacy, the combination of a selective IDO1 inhibitor and pembrolizumab in unselected melanoma patients failed to improve outcomes in a phase 3 randomized study [133, 134]. Recently, Li and colleagues [135] conducted a comprehensive study of the Trp-Kyn pathway in melanoma and RCC patients treated with nivolumab. In this study, treatment with PD-1 blockade induced Trp/Kyn conversion. More importantly, the increase of the Kyn/Trp ratio during treatment was a predictor of survival in both cohorts of melanoma and RCC, and it was further validated in a larger independent study comparing nivolumab vs. everolimus in pretreated RCC patients. At week 4, the Kyn/Trp increase was significantly associated with worse OS in the nivolumab arm but not in the everolimus arm (Figure 1). These results suggest that serum Kyn/Trp monitoring could help identify which patients are more likely to benefit from IDO and PD-1 inhibition and deserve further study.

## Conclusions

Current research on circulating biomarkers for predicting response to ICI treatment in RCC is promising, although it is still in its early stages (Figure 1). In addition, circulating biomarkers have not been extensively studied in the context of ICI combination therapy in aRCC, the standard of care in first-line setting. However, these biomarkers, encompassing immune cell populations, soluble factors, ctDNA, CTCs, and metabolomic profiles, hold significant potential. Compared to tissue biopsies, they are less invasive, potentially more comprehensive, and may enable real-time monitoring. The analysis of immune cell populations and circulating soluble factor seems to provide valuable insights into the immune landscape and real-time monitoring that could help identify patients who are resistant or responsive to ICI. Meanwhile circulating DNA and CTCs could help guide treatment selection and de-escalation/intensification strategies, by idenfitying patients at higher risk of recurrence and by discerning good from poor responders. Circulating biomarkers hold the potential to help us identify which patients will benefit most from double ICI therapy or ICI-AA therapy.

To harness these potentials, it is essential to standardize detection methods and establish consistent cut-offs, and validate these biomarkers in large, randomized clinical trials. Moreover, achieving meaningful predictive accuracy will likely require the integration of multiple biomarkers and multiomics techniques. Studies like the pragmatic European CARE1 trial, which evaluates double ICI against ICI-AA therapy, are incorporating integrated circulating biomarkers studies that could provide new insights. Integration of biomarker studies into clinical trials will be essential for advancing these biomarkers towards clinical practice.

## Abbreviations

AA: anti-angiogenic
aRCC: advanced renal cell carcinoma
BAFF: B-cell activating factor
CA: cancer antigen
ccRCC: clear cell renal cell carcinoma
cfDNA: cell-free deoxyribonucleic
CR: complete response
CTCs: circulating tumor cells
ctDNA: circulating tumor deoxyribonucleic
DCR: disease control rate
DFS: disease-free survival
DNA: deoxyribonucleic
EV: extracellular vesicle
ICI: immune checkpoint inhibitors
IDO: indoleamine 2,3-dioxygenase
IFN-γ: interferon gamma
KIM-1: Kidney-Injury Molecule-1
Kyn: kynurenine
LIPI: lung immune prognostic index
miRNAs: micro-ribonucleic acids
mRCC: metastatic renal cell carcinoma
MUC1: mucin 1
NER: neutrophil-to-eosinophil ratio
NLR: neutrophil-to-lymphocyte ratio
NSCLC: non-small cell lung cancer
OR: odds ratio
OS: overall survival
PFS: progression-free survival
PLR: platelet-to-lymphocyte ratio
PR: partial response
RCC: renal cell carcinoma
sCD27: soluble CD27
SII: systemic immune inflammation
sPD-L1: soluble PD-L1
TCRB: T-cell receptor β-chain
TME: tumor microenvironment
Trp: tryptophan
UC: urothelial carcinoma
VEGF: vascular-endothelial growth factor

## Declarations

### Copyright

© The Author(s) 2024.
